# Retracted: Herbal Supplement Ameliorates Cardiac Hypertrophy in Rats with CCl_4_-Induced Liver Cirrhosis

**DOI:** 10.1155/2017/5276749

**Published:** 2017-11-22

**Authors:** Evidence-Based Complementary and Alternative Medicine

Evidence-Based Complementary and Alternative Medicine has retracted the article titled “Herbal Supplement Ameliorates Cardiac Hypertrophy in Rats with CCl_4_-Induced Liver Cirrhosis” [1]. The article was found to contain duplicated images in Figure 4(a), in which the IL6 blots are identical to the p-ERK5 blots. The authors provided a corrected figure, with replacements for p-ERK5, which is available as Supplementary Materials. However, they could not provide the underlying blots.

## References

[1] Li P.-C., Chiu Y.-W., Lin Y.-M. (2012). Herbal supplement ameliorates cardiac hypertrophy in rats with CCl_4_-induced liver cirrhosis. *Evidence-Based Complementary and Alternative Medicine*.

